# More than just anti-NMDAR: the many facets of autoimmune encephalitis

**DOI:** 10.1192/bjb.2021.113

**Published:** 2022-08

**Authors:** Karim Abdel Aziz, Emmanuel Stip, Danilo Arnone

**Affiliations:** 1 College of Medicine and Health Sciences, United Arab Emirates University, Al-Ain, UAE; 2 Institute Universitaire en Santé Mentale de Montréal, Université de Montréal, Canada; 3 Institute of Psychiatry, Psychology and Neuroscience, King's College London, UK

**Keywords:** Anti-NMDAR encephalitis, autoimmune encephalitis, neuropsychiatry, NMDA receptors, autoimmune encephalitides

## Abstract

This editorial expands on a Praxis article published by Beattie and colleagues in the trainees’ section of this journal. The authors describe an interesting case of anti-*N*-methyl-d-aspartate receptor (anti-NMDAR) encephalitis, outline the clinical presentation and make suggestions on ways to approach this rare disorder. Here we provide an overview of autoimmune conditions that result in the production of autoantibodies targeting central nervous system proteins mediating autoimmune encephalitis and offer a perspective on approaches to diagnosis and treatment.

In their Praxis article, Beattie and colleagues present a case of anti-*N*-methyl-d-aspartate receptor (anti-NMDAR) encephalitis and make suggestions for psychiatrists on how to approach such difficult clinical situations.^1^ The importance of this topic to psychiatrists lies in the fact that although anti-NMDAR encephalitis is primarily a neurological disorder, nearly 80% of these patients first present with psychiatric symptoms and more than 60% are first admitted to psychiatric units.^2^ It is estimated that nearly 75% of these individuals recover or have minimal deficits, although up to 25% endure severe functional deficits or even die, mostly owing to delays in diagnosis.^3^ Hence, early recognition and treatment are key to a successful outcome.

The clinical case described by Beattie and colleagues is of a woman in her mid-20s treated initially for a psychiatric disorder who later developed neurological deficits raising suspicion of anti-NMDAR encephalitis. The authors demonstrate the challenges likely to occur in clinical situations when there is the potential for being misled by the initial emergence of psychiatric symptoms as early manifestation of this disease. Following a systematic approach to clinical reasoning might help to consider the possibility of anti-NMDAR encephalitis as early as possible.^4^ Early cues are an important element in increasing the chance of early diagnosis of anti-NMDAR encephalitis, essential to improve outcome. Beattie and colleagues highlight the importance of ‘red flags’ to aid the diagnosis of autoimmune encephalitis. These red flags include a host of associated manifestations that could aid early diagnosis, especially when detecting for the presence of autoantibodies, central to diagnosis and treatment of anti-NMDAR encephalitis, is not feasible. This editorial provides a succinct description of the several types of autoantibodies associated with autoimmune encephalitis and highlights the difficulties in reaching the diagnosis and providing treatment.

## Autoimmune encephalitis and autoantibody subtypes

Anti-NMDAR encephalitis is the most frequently occurring of several types of autoimmune encephalitis.^5^ It affects around 1.5 per million people/year.^6^ Autoimmune encephalitides are broadly divided into two categories according to whether the immunological mechanism involves autoantibodies targeting ‘extracellular’ or ‘intracellular’ neuronal antigens. The overall incidence has increased from 0.4/100 000 in 1995–2005 to 1.2/100 000 in 2006–2015.^7^ This could be explained by changes in consensus definitions, which initially focused on neuronal surface autoantibody-mediated encephalopathies and only subsequently included paraneoplastic limbic and anti-voltage-gated potassium channel antibodies.

There is a crucial distinction between paraneoplastic syndromes associated with onconeural antibodies and other autoimmune encephalopathies. Onconeural antibodies against surface antigenic targets are in fact biomarkers with no bearing on the disease process, unlike the more common autoimmune encephalitides. Onconeural antibodies against intracellular targets represent malignancy.

Autoantibodies that often target extracellular receptors and ion channels include those for NMDA (*N*-methyl-d-aspartate), AMPA (α-amino-3-hydroxy-5-methyl-4-isoxazolepropionic acid), GABA (gamma-aminobutyric acid), glycine receptors, and voltage-gated potassium channels (VGKC) including LGI1 (leucine-rich glioma inactivated 1) and CASPR2 (contactin-associated protein-like 2) proteins.^8^ Autoantibodies that target either intracellular antigens or synaptic proteins include anti-Hu, anti-Ri, anti-Yo, anti-Ma, anti-amphiphysin and anti-GAD (glutamic acid decarboxylase). The immune response that targets intracellular antigens is thought to involve CD8+ cytotoxic T-cell-mediated cell injury after binding of autoantibodies to the target intracellular antigen. These autoimmune encephalitides are often paraneoplastic manifestations of various types of cancer.^9,10^

Although psychiatric symptomatology can occur with any antibody-positive encephalitis, it is more frequent in presentations associated with autoantibodies targeting extracellular antigens.^11^

### Autoantibodies targeting extracellular antigens

#### Anti-NMDAR encephalitis

NMDA receptors are glutamatergic ionotropic receptors often found in the presynaptic GABA neurons of the thalamus and frontal lobes. Impairment of these receptors leads to dysfunction of glutamatergic and dopaminergic networks throughout the brain.^8^

Animal studies suggest that anti-NMDA receptors passively transferred into the brains of rodents produce neurological symptoms proportionate to the surface reduction of NMDA receptors on the neurons,^12^ causing depletion of NMDA receptors from the synapse.^13^ This depletion can explain the insurgence of psychotic symptoms experienced by patients (early confusion, psychosis, visual hallucinations and personality change, followed by neurological deficits),^9^ which is analogous to psychotic manifestations observed with phencyclidine ingestion, which also acts through NMDA receptor hypofunction.^10^

#### Anti-AMPAR encephalitis

AMPA receptors are also glutamatergic ionotropic receptors that are widely expressed in the brain mediating rapid excitatory transmission.^10^ Anti-AMPAR encephalitis tends to present with psychiatric symptoms similar to anti-NMDAR encephalitis. The median age at onset of this type of encephalitis tends to be in the 40s and 50s,^8,14^ which differs from anti-NMDAR encephalitis, which mostly affects females in their 20s.^8^

#### Anti-LG1 antibody encephalitis

LGI1 is a presynaptic glycoprotein involved in the regulation of AMPA and VGKC receptors. On cultured neurons, LGI1 antibodies have been shown to affect AMPA receptor localisation across the whole brain. Classic symptoms of anti-LGI1 antibody encephalitis include hyponatraemia and faciobrachial dystonic seizures with initial subtle memory loss and sleep disorders (hypersomnia, insomnia, rapid-eye movement (REM) sleep behaviour disorder, sleep reversal). This condition tends to occur at a median age of 60 years.^9,15^

#### CASPR2 antibody encephalitis

CASPR2 is a cell adhesion molecule that organises VGKCs in the central and peripheral nervous system. People with anti-CASPR2 antibodies develop symptoms originating in the central nervous system (limbic encephalitis) and/or the peripheral nervous system (e.g. Morvan syndrome), with encephalitis characterised by confusion, amnesia and hallucinations. Onset is usually slower than for anti-NMDAR encephalitis and the median age at onset is 60 years.^10,16^

#### Anti-GABA receptor encephalitis

GABA is the primary inhibitory ionotropic receptor in the brain. Antibodies targeting GABA_A_ and GABA_B_ receptors result in a type of limbic encephalitis associated with severe seizures that can lead to status epilepticus, together with psychiatric manifestations such as memory loss, confusion, hallucinations and personality change.^17–19^

#### Glycine-receptor autoantibody-associated disease

The glycine receptor is an inhibitory receptor. The α_1_ subunit is the predominant antigenic target.^20^ Studies involving the transfer of human glycine autoantibodies to rodents have demonstrated that neural transmission at the cellular level with consecutive, altered signal cascades induces psychiatric and cognitive symptoms. Glycine-receptor antibodies have been found in ‘stiff person spectrum’ disorders such as progressive encephalomyelitis with rigidity and myoclonus and have been associated with cognitive/memory dysfunction and psychosis.^11^

### Autoantibodies targeting intracellular antigens and proteins

Autoantibodies that target intracellular antigens (anti-Hu, anti-Ri, anti-Ma, anti-Yo) and proteins (anti-amphiphysin, anti-GAD) typically present as paraneoplastic manifestations. Neuropsychiatric symptoms often precede the diagnosis of the primary neoplasia by several months. Common symptoms include depression, irritability, hallucinations, sleep disturbance and seizures. Memory loss can occur over weeks to months, in some cases progressing to severe cognitive decline resembling a dementing illness.^9^ The latter has been associated with anti-Hu antibodies, which target an intracellular RNA-packed protein, important in memory-related synaptic plasticity.^11^ Anti-Ri antibodies have been linked with lung carcinoma. Typical manifestations include personality changes and neuropsychological deficits.^11^ Anti-Ma antibodies are often found in young males with germ cell tumours and are associated with a range of psychiatric manifestations, including obsessive–compulsive disorder, delirium, major depression, personality changes and amnesia.^11^ Anti-Yo antibodies are mostly found in females with breast or ovarian cancers. These antibodies most likely act through T-cell mechanisms and are associated with psychosis.^11,21,22^

Anti-amphiphysin antibodies are very strongly associated with breast cancer in women. These antibodies target intracellular proteins important for recycling synaptic vesicles. Animal models suggest that in rats, when anti-amphiphysin antibodies are passively transferred intrathecally they can induce anxiety behaviours.^10,23^ Anti-GAD antibodies target the synaptic isoform of the enzyme necessary to synthesise GABA.^10^ Mood changes and cognitive impairment are the most frequent symptoms.^11^

### Summary

Although anti-NMDAR encephalitis is the most common of the autoimmune encephalitides, there are several other types of autoantibody targeting extracellular and intracellular antigens that can present with similar clinical characteristics. In the presence of atypical, often rapidly evolving psychiatric manifestations, especially when coexisting with neurological symptoms, an early age at onset in a female patient or a mid to late onset in both genders might raise the suspicion of encephalitis. It is important to consider the possibility of a primary neoplasia and a relatively recent diagnosis of cancer, especially if originating in the ovaries, breast, lung and germ cells.

## Autoantibody screening

Beattie and colleagues highlight the complexities of screening and interpreting serum/cerebrospinal fluid (CSF) anti-NMDAR autoantibody levels in patients presenting with psychiatric symptoms. Rates of false positives and false negatives in affected individuals are high, in the range of 10%.^24,25^

Commercial kits are now available for different types of autoantibody other than anti-NMDAR (e.g. LGI1, Caspr2, AMPAR and GABA),^10^ although results may require several weeks of processing. For extracellular antibody tests other than anti-NMDAR encephalitis, CSF is still the most sensitive and specific test, with serum offering a high rate of false-negative results.^10^ In the case of intracellular antibody tests, positive test results on their own might not be sufficient as definitive confirmation of a particular autoimmune aetiology. Some of these autoantibodies, for example anti-GAD65, may be associated with other disorders (e.g. type 1 diabetes) and may coexist with other autoantibodies, for example anti-GABA_B_.

A recent meta-analysis suggests that in cross-sectional studies NMDAR IgG antibodies are more common in people with psychosis than in controls^26^ and a further recent study showed the limitations of commercial assays as diagnostic tests for autoimmune encephalitis.^27^ Excessive screening based on recognition of the stereotyped clinical syndromes but without sufficient prior probability is controversial. Moreover, although pairing serum with CSF testing can be helpful in correlation with clinical symptoms, it is important to recognise that in some autoimmune encephalitides (e.g. LGI1), autoantibodies are often undetectable in CSF. Furthermore, as these conditions differ epidemiologically (e.g. LGI1 and CASPR2 are exceedingly rare in young people, and NMDAR is rare in older adults) a targeted approach to testing is greatly advisable.

In summary, depending on the laboratory and assay used, autoantibodies can be detected outside of the canonical clinical syndromes and overinterpreting these results can cause harm. In view of this uncertainty and the possibility that immunotherapy might be effective, the recent consensus is to consider a diagnosis of ‘seronegative autoimmune encephalitis’ in the presence of clinical symptoms and absence of autoantibodies.^28^

## Implications for diagnosis and treatment

Diagnostic uncertainty, highly disturbed mental states, logistic difficulties in carrying out a range of essential investigations (lumbar puncture, electroencephalogram and magnetic resonance imaging) can delay the prospective identification of these conditions, particularly in undifferentiated psychiatric services. The use of screening criteria for anti-NMDAR encephalitis can help improve the detection of autonomic dysfunction, cognitive impairment and movement disorders, but ‘red flag’ symptoms tend to overlap with those seen in functional psychiatric illness.^29^

Currently there is no definitive treatment algorithm for anti-NMDAR encephalitis. Recommended initial therapy includes intravenous immunoglobulins and methylprednisolone or daily plasma exchange. For refractory illness, second-line treatments include rituximab and cyclophosphamide. The proteasome inhibitor bortezomib has shown some efficacy in highly refractory disease.^30,31^ Similar immunosuppressant strategies can be used for the other types of extracellular autoimmune encephalitis. Anti-LGI1 encephalitis generally responds well to first-line treatment,^28^ and rituximab is thought to be generally effective where the autoantibodies are of the IgG4 subtype, which predominate in anti-LGI1 and anti-CASPR2 encephalitides.^10^

Patients with clinical symptoms due to autoantibodies targeting intracellular antigens are believed to respond poorly to immunotherapy. This is possibly related to CD8+ mediated cytotoxicity, which may cause irreversible neuronal cell injury. For these patients prompt detection and treatment of the underlying neoplasm is the best approach for symptom resolution.^28^

In summary**,** there is variability in the approach to diagnosis and treatment of autoimmune encephalitis often driven by the specialty of the assessing clinician rather than the clinical presentation. Hence, for suspected cases there is great need to collaborate with local neurologists to establish preferential diagnostic pathways involving regional neuroscience centres where systematic diagnostic assessment and investigations can be facilitated. Immunosuppressant strategies are considered the most effective treatment of extracellular types of autoimmune encephalitis, whereas identifying and treating the cancerous source of paraneoplastic symptoms is the most effective approach for the type of autoimmune encephalitis targetting intracellular antigens.

## Conclusions

The presence of autoantibodies against brain receptors or proteins can result in severe yet potentially treatable autoimmune encephalitis. Detecting autoantibodies is a necessary but not always informative diagnostic step. History or suspicion of cancer should alert clinicians. Atypical psychiatric manifestations, commonly associated with neurological symptoms, raise concerns about the origin of these disorders. The clinical distinction between autoimmune encephalitis and psychiatric presentations is very challenging. The use of screening tools and preferential diagnostic pathways in collaboration with local neurologists with access to regional neuroscience centres could facilitate reaching a timely diagnosis.

## Data Availability

Data availability is not applicable to this article as no new data were created or analysed in this study.

## About the authors

**Karim Abdel Aziz** is an Associate Professor in the Department of Psychiatry, College of Medicine and Health Sciences, United Arab Emirates University (UAEU), Al-Ain, UAE. **Emmanuel Stip** is a Professor and Chair of the Department of Psychiatry, College of Medicine and Health Sciences, UAEU, Al-Ain, UAE; he is also Professor Emeritus of Psychiatry in the Department of Psychiatry, Institute Universitaire en Santé Mentale de Montréal, Université de Montréal, Canada. **Danilo Arnone** is an Associate Professor in the Department of Psychiatry, College of Medicine and Health Sciences, UAEU, Al-Ain, UAE; he is also affiliated with the Centre for Affective Disorders, Institute of Psychiatry, Psychology and Neuroscience, King's College London, UK.

## References

[1] Beattie M, Goodfellow J, Oto M, Krishnadas R. (2021) Anti-NMDAR encephalitis for psychiatrists: the essentials. BJPsych Bulletin [Epub ahead of print] 2 Jun 2021. Available from: (10.1192/bjb.2021.35 [cited 21 Mar 2019]). PMC9768510 34075874

[2] Bost C, Pascual O, Honnorat J. Autoimmune encephalitis in psychiatric institutions: current perspectives. Neuropsychiatr Dis Treat 2016; 12: 2775–87. 27822050 10.2147/NDT.S82380 PMC5089825

[3] Dalmau J, Gleichman AJ, Hughes EG, Rossi JE, Peng X, Lai M, Anti-NMDA-receptor encephalitis: case series and analysis of the effects of antibodies. Lancet Neurol 2008; 7: 1091–8. 18851928 10.1016/S1474-4422(08)70224-2 PMC2607118

[4] Abdel Aziz K, AlSuwaidi A, Al-Ammari A, Al Khoori A, AlBloushi A, Al-Nuaimi N, When should psychiatrists think of anti-NMDA receptor encephalitis? A systematic approach in clinical reasoning. Asian J Psychiatr 2021; 56: 102524. 33418282 10.1016/j.ajp.2020.102524

[5] Huang YQ, Xiong H. Anti-NMDA receptor encephalitis: a review of mechanistic studies. Int J Physiol Pathophysiol Pharmacol 2021; 13(1): 1–11. 33815666 PMC8012859

[6] Dalmau J, Armangué T, Planagumà J, Radosevic M, Mannara F, Leypoldt F, An update on anti-NMDA receptor encephalitis for neurologists and psychiatrists: mechanisms and models. Lancet Neurol 2019; 18: 1045–57. 31326280 10.1016/S1474-4422(19)30244-3

[7] Dubey D, Pittock SJ, Kelly CR, McKeon A, Lopez-Chiriboga AS, Lennon VA, Autoimmune encephalitis epidemiology and a comparison to infectious encephalitis. Ann Neurol 2018; 83: 166–77. 29293273 10.1002/ana.25131 PMC6011827

[8] Dalmau J, Graus F. Antibody mediated encephalitis. New Eng J Med 2018; 378: 840–51. 29490181 10.1056/NEJMra1708712

[9] Deng P, Yeshokumar A. Autoimmune encephalitis. What psychiatrists need to know. Psychiatr Times 2020; 27(1): 7–9.

[10] Lancaster E. The diagnosis and treatment of autoimmune encephalitis. J Clin Neurol 2020; 12(1): 1–13. 10.3988/jcn.2016.12.1.1 PMC4712273 26754777

[11] Hansen N, Timäus C. Autoimmune encephalitis with psychiatric features in adults: historical evolution and prospective challenge. J Neural Transm 2021; 128(1): 1–14. 33026492 10.1007/s00702-020-02258-z PMC7815593

[12] Planagumà J, Leypoldt F, Mannara F, Gutiérrez-Cuesta J, MartínGarcía E, Aguilar E, Human *N*-methyl D-aspartate receptor antibodies alter memory and behaviour in mice. Brain 2015; 138: 94–109. 25392198 10.1093/brain/awu310 PMC4285189

[13] Moscato EH, Jain A, Peng X, Hughes EG, Dalmau J, Balice-Gordon RJ. Mechanisms underlying autoimmune synaptic encephalitis leading to disorders of memory, behavior and cognition: insights from molecular, cellular and synaptic studies. Eur J Neurosci 2010; 32: 298309. 10.1111/j.1460-9568.2010.07349.x PMC2955837 20646055

[14] Lai M, Hughes EG, Peng X, Zhou L, Gleichman AJ, Shu H, AMPA receptor antibodies in limbic encephalitis alter synaptic receptor location. Ann Neurol 2009; 65: 424–34. 19338055 10.1002/ana.21589 PMC2677127

[15] Ohkawa T, Fukata Y, Yamasaki M, Miyazaki T, Yokoi N, Takashima H, Autoantibodies to epilepsy-related LGI1 in limbic encephalitis neutralize LGI1-ADAM22 interaction and reduce synaptic AMPA receptors. J Neurosci 2013; 33: 18161–74. 24227725 10.1523/JNEUROSCI.3506-13.2013 PMC3828467

[16] Dalmau J, Rosenfeld MR. Autoimmune encephalitis update. Neuro Oncol 2014; 16: 771–8. 24637228 10.1093/neuonc/nou030 PMC4022229

[17] Lancaster E, Lai M, Peng X, Hughes E, Constantinescu R, Raizer J, Antibodies to the GABA(B) receptor in limbic encephalitis with seizures: case series and characterisation of the antigen. Lancet Neurol 2010; 9: 67–76. 19962348 10.1016/S1474-4422(09)70324-2 PMC2824142

[18] Petit-Pedrol M, Armangue T, Peng X, Bataller L, Cellucci T, Davis R, Encephalitis with refractory seizures, status epilepticus, and antibodies to the GABA A receptor: a case series, characterisation of the antigen, and analysis of the effects of antibodies. Lancet Neurol 2014; 13: 276–86. 24462240 10.1016/S1474-4422(13)70299-0 PMC4838043

[19] Höftberger R, Titulaer MJ, Sabater L, Dome B, Rózsás A, Hegedus B, Encephalitis and GABAB receptor antibodies: novel findings in a new case series of 20 patients. Neurology 2013; 81: 1500–6. 24068784 10.1212/WNL.0b013e3182a9585f PMC3888170

[20] Martinez-Hernandez E, Arino H, McKeon A, Iizuka T, Titulaer MJ, Simabukuro MM, Clinical and immunologic investigations in patients with stiff-person spectrum disorder. JAMA Neurol 2016; 73: 714–20. 27065452 10.1001/jamaneurol.2016.0133 PMC5020136

[21] Tanaka K, Kawamura M, Sakimura K, Kato N. Significance of autoantibodies in autoimmune encephalitis in relation to antigen localization: an outline of frequently reported autoantibodies with a non-systematic review. Int J Mol Sci 2020; 21: 4941. 32668637 10.3390/ijms21144941 PMC7404295

[22] Greenlee JE, Clawson SA, Hill KE, Wood BL, Tsunoda I, Carlson NG. Purkinje cell death after uptake of anti-Yo antibodies in cerebellar slice cultures. J Neuropathol Exp Neurol 2010; 69: 997–1007. 20838245 10.1097/NEN.0b013e3181f0c82b PMC2959164

[23] Geis C, Grünewald B, Weishaupt A, Wultsch T, Toyka KV, Reif A, Human IgG directed against amphiphysin induces anxiety behaviour in a rat model after intrathecal passive transfer. J Neural Transm 2012; 119: 981–5. 22331304 10.1007/s00702-012-0773-3

[24] Hammer C, Stepniak B, Schneider A, Papiol S, Tantra M, Begemann M, Neuropsychiatric disease relevance of circulating anti-NMDA receptor autoantibodies depends on blood–brain barrier integrity. Mol Psychiatry 2014; 19: 1143–9. 23999527 10.1038/mp.2013.110

[25] Gresa-Arribas N, Titulaer MJ, Torrents A, Aguilar E, McCracken L, Leypoldt F, Antibody titres at diagnosis and during follow-up of anti-NMDA receptor encephalitis: a retrospective study. Lancet Neurol 2014; 13: 167–77. 24360484 10.1016/S1474-4422(13)70282-5 PMC4006368

[26] Cullen AE, Palmer-Cooper EC, Hardwick M, Vaggers S, Crowley H, Pollak TA, Influence of methodological and patient factors on serum NMDAR IgG antibody detection in psychotic disorders: a meta-analysis of cross-sectional and case-control studies. Lancet Psychiatry 2021; 8: 109–20. 33357497 10.1016/S2215-0366(20)30432-6

[27] Ruiz-García R, Muñoz-Sánchez G, Naranjo L, Guasp M, Sabater L, Saiz A, Limitations of a commercial assay as diagnostic test of autoimmune encephalitis. Front Immunol 2021; 12: 691536. 34267758 10.3389/fimmu.2021.691536 PMC8276168

[28] Ellul MA, Wood G, Tooren H, Easton A, Babu A, Michael BD. Update on the diagnosis and management of autoimmune encephalitis. Clin Med 2020; 20: 389–92. 10.7861/clinmed.2020-0241 PMC7385764 32675144

[29] Warren N, Flavell J, O'Gorman C, Swayne A, Blum S, Kisely S, Screening for anti-NMDAR encephalitis in psychiatry. J Psychiatr Res 2020; 125: 28–32. 32203736 10.1016/j.jpsychires.2020.03.007

[30] Titulaer MJ, McCracken L, Gabilondo I, Armangué T, Glaser C, Iizuka T, Treatment and prognostic factors for long-term outcome in patients with anti-NMDA receptor encephalitis: an observational cohort study. Lancet Neurol 2013; 12: 157–65. 23290630 10.1016/S1474-4422(12)70310-1 PMC3563251

[31] Simmons ML, Perez KA. Bortezomib for treatment of anti–NMDA receptor encephalitis in a pediatric patient refractory to conventional therapy. Am J Health Syst Pharm 2021; 78: 395–400. 33547464 10.1093/ajhp/zxaa415

